# Pleomorphic Adenoma of Nasal Septum a Case Report

**DOI:** 10.1007/s12070-023-03530-w

**Published:** 2023-02-10

**Authors:** Guangjun Tang, Bo Du, Youxing Lan, Li Tian

**Affiliations:** 1 Department of Otolaryngology, Hospital of Chengdu University of Traditional Chinese Medicine, No. 39 of Shi’erqiao Road, Jinniu District, 610075, 610072 Chengdu, Chengdu, China; 2 Department of otolaryngology head and neck surgery, People’s Hospital of Anshun City Guizhou Province, No. 140 of Huangguoshu Street, Xixiu District, 561000 Guizhou, Anshun City, Guizhou Province China

**Keywords:** Pleomorphic Adenoma, case report, nasal septum

## Abstract

**Introduction:**

Pleomorphic adenoma is a common benign tumor of large and small salivary glands. It mainly occurs in the parotid gland, followed by the submandibular gland, sublingual gland and small salivary gland in the oral cavity. It is very rare in the nasal septum.

**Patient Concerns:**

A 27-year-old female patient attended our clinic with Nasal congestion and a diminished sense of smell.

**Diagnoses:**

Endoscopic examination revealed a mass within the right nasal passage. A pathological biopsy revealed pleomorphic adenoma.

**Interventions:**

The nasal septum pleomorphic adenoma was resected by endoscopic approach.

**Outcomes:**

No recurrence was observed for over 41 months of follow-up.

**Conclusion:**

To prevent recurrence, extensive local resection with clear histological margins and long-term endoscopic follow-up with an endoscope are necessary.

## Introduction

Pleomorphic adenoma is a benign tumor commonly seen in both large and small salivary glands. It is composed of a mixture of epithelial and mesenchymal tissues. It mainly occurs in parotid gland, followed by submandibular gland, sublingual gland and minor salivary gland in oral cavity, but in nasal septum is very rare [1–4].

## Case Report

**Fig. 1:**
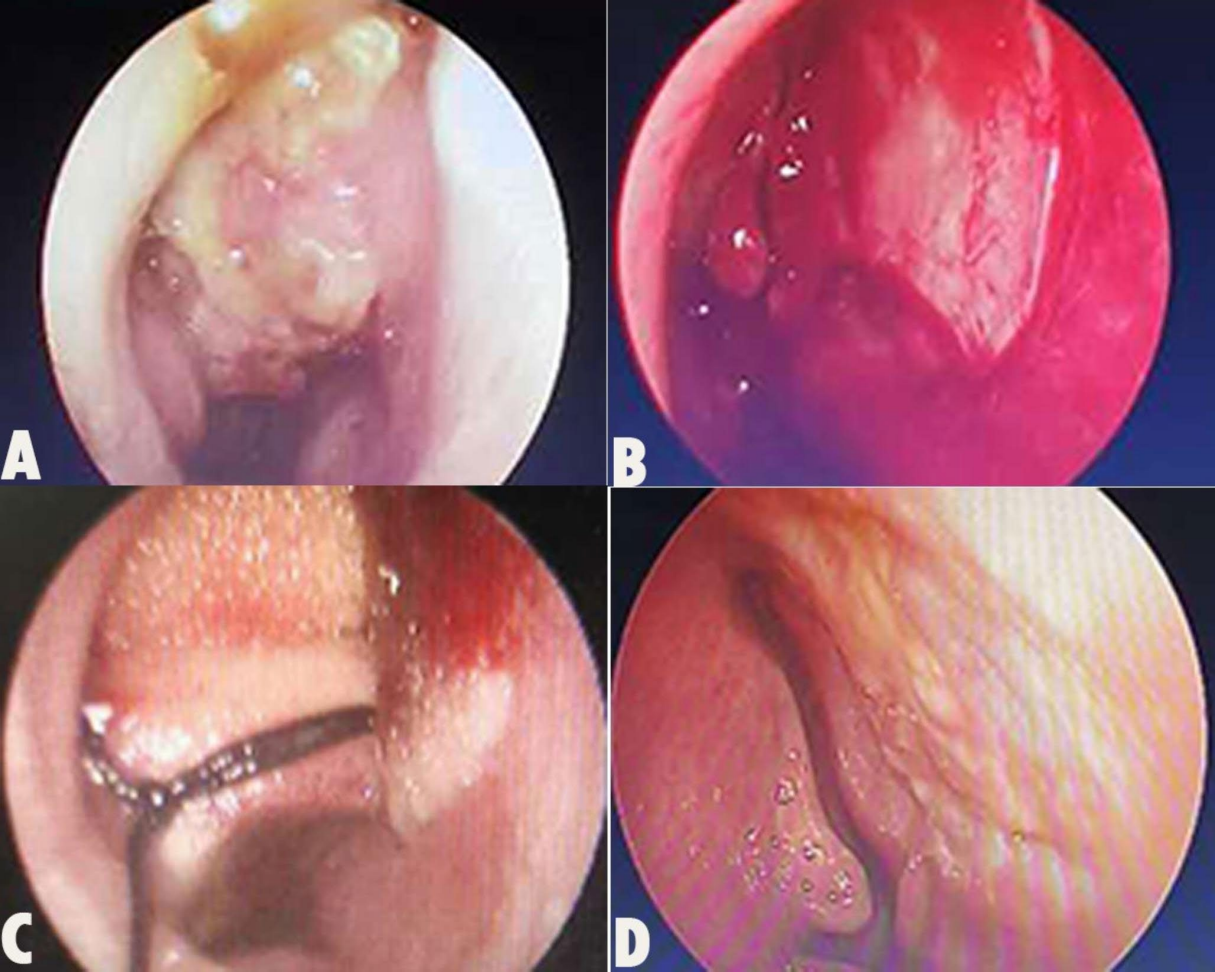
A: Endoscopic examination revealed an elastic hard, oval, and mucosa-covered mass (2.5 cm x 2.1 cm), was located in her posterior upper part of the right nasal septum with a broad base; B and C was the postoperation; D: Follow-up was observed at 2 month

**Fig. 2:**
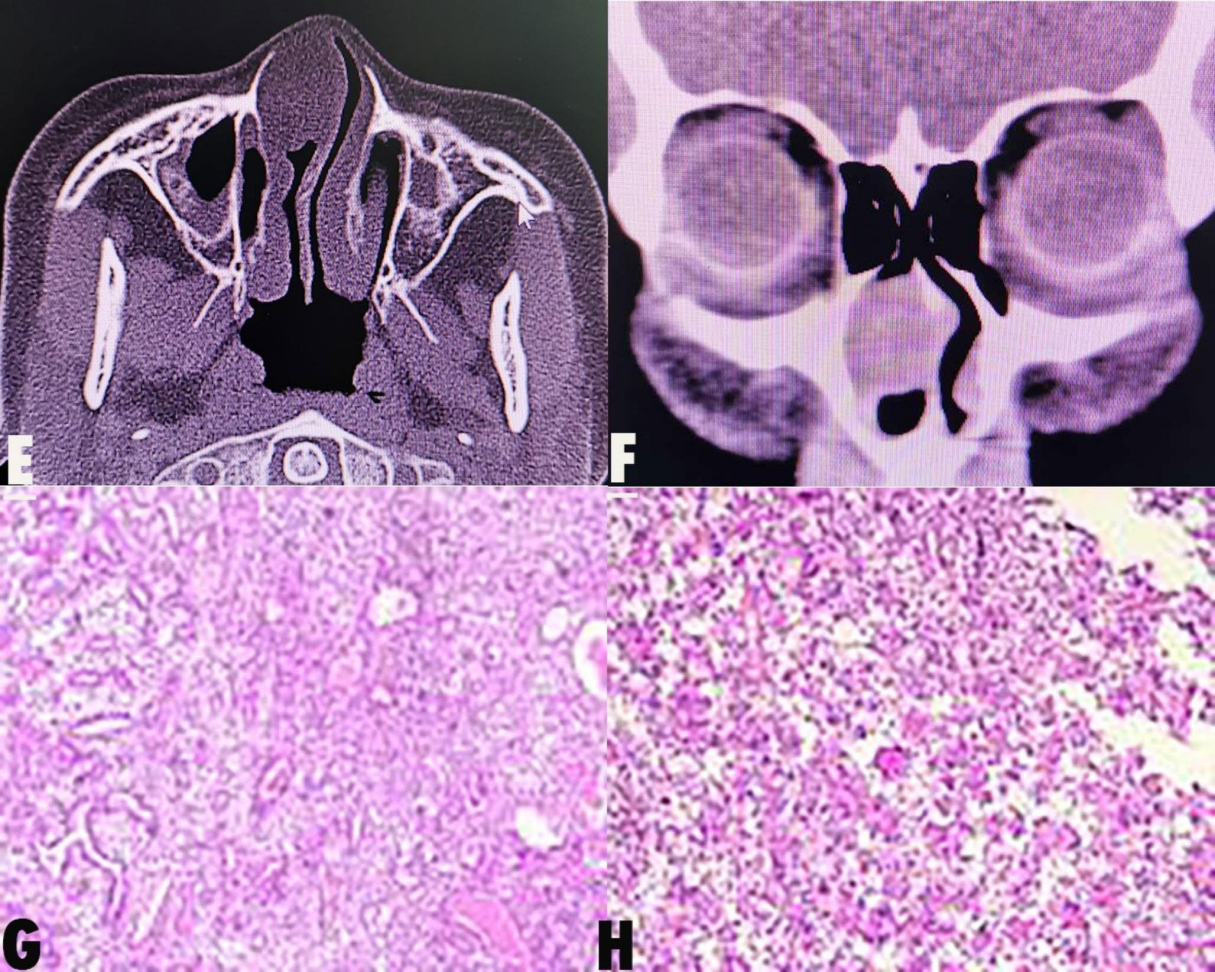
E and F suggested that a CT scan revealed an oval mass with soft-density in the right nasal cavity; G and H are the graphs of preoperative pathological biopsy report

We report a case of a Pleomorphic adenoma of nasal septum (PANS) of a 27-year-old woman who presented with a 1- month history of right nasal congestion, rhinnorhea, accompanied by hyposmia. She had no particular past or family history of illness. Endoscopic examination revealed a mass within the right nasal cavity (Fig. 1). On physical examination, there was an elastic hard, oval, and mucosa-covered mass in her posterior upper part of the nasal septum with a broad base. Computed tomographic scans (CT) revealed an oval mass in the right nasal cavity with soft-density of about 2.5 cm x 2.1 cm (Fig. 2).

A biopsy of the mass was performed for pathological examination. Clinical and histopathological examinations showed that the mass is a pleomorphic adenoma of nasal septum (Fig. 2). On June 24, 2019, Surgical treatment was performed with the consent of the patient and there were no obvious contraindications in preoperative examinations. After local infiltration and anesthesia of the nasal cavity with the assistance of nasal endoscope, completely removed the mass with 5 mm outside margin of the base in the nasal septum mucosa of right nasal cavity.

**Fig. 3:**
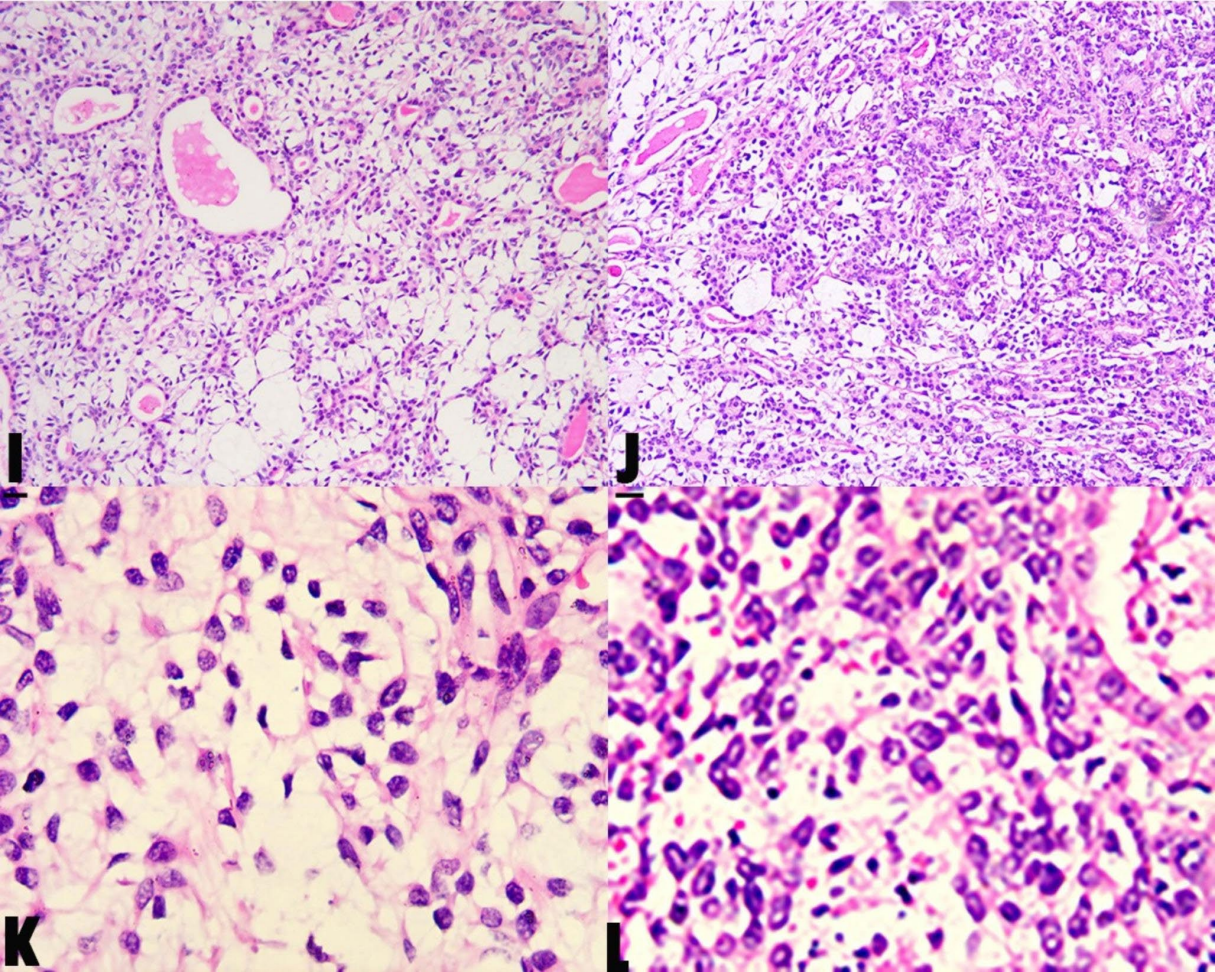
**HE ­ 100X - I - J**: This is a pleomorphic adenoma dominated by myoepithelial cells. Microscopically, it can be seen that small fat spindle cells are distributed in sheets under the background of mucus, loosely arranged, and part of the glandular tubular structure. A few cartilaginous areas are seen **HE ­ 400X - K - L**: The short columnar and cubic glandular epithelium forms a double-layer glandular tubular structure. The inner layer is surrounded by glandular epithelium and the outer layer is composed of myoepithelial cells.The myoepithelial cells are small fusiform and small round

The nasal septum mucosa and periosteum margins bounded by the base of the mass were electrocoagulated with low temperature plasma. The excised mass was biopsied. Clinical and histopathological examination revealed that the mass was a pleomorphic adenoma which originated in nasal septum (Fig. 3). At the second and 41th month follow-up, there was no recurrence.

## Discussion

Pleomorphic Adenoma is a benign tumor, the histology is derived from leap tube epithelium of salivary glands. it is also called a mixed adenomatosis due to its tendency towards multidirectional differentiation (myoepithelial differentiation and glandular epithelium differentiation). They are mainly distributed in the major salivary gland, of which about 85% occur in the parotid gland, 8% in the submandibular gland, and about 7% in the minor salivary gland [5]. Which includes the hard and soft palate, the nasal cavity, pharynx, larynx, trachea, and lacrimal glands [1]. The clinical incidence of septum is rare.

Pleomorphic adenoma which originates from the mucosa of nasal septum have the different hypotheses [6]. Matthew et al. suggested that the abnormal origin of these tumors may come from the mucosa of the nasal septum, possibly due to the misplacement of embryonic epithelial cells, which come from the epidermis and enter the nasal septum through the septal region. According to Stevenson pointed out, the tumors in this area originates from a remnant of the vomeronasal organ, an epithelium-lined duct in the septum that degenerated in early fetal life. Conversely, Evans and Cruickshank state that, at present, mixed neoplasms are almost universally believed to originate entirely from epithelial cells and occur in fully developed salivary gland tissues rather than embryonic remains [7].

Pleomorphic Adenoma is usually unilateral nasal diseases, growth slow, mostly expansive growth. There are different clinical features according to the tumor progression and size [2, 3, 7–9]. In the early stages, the tumor is small, may not have obvious clinical manifestations, and is often diagnosed by physical examination or incidental discovery. As the tumor continues to grow, the nasal passages are blocked, it can be present as a unilateral nasal congestion, progressive aggravation, and accompanied by nose bleeding or headache, smell disorders and so on. If the tumor expands further and grows along the nasal space, it often leads to poor sinus drainage, sinusitis or mucosal edema, and even polyps. At the same time, the surface of the enlarged tumor envelope may become necrotic and form granulation. Swelling, pain and deformities may occur in the nose or face if the surrounding tissues are involved. If the dacryocyst and orbit are violated, tears overflow, eyeball displacement, and diplopia may occur. Physical examination showed that the tumors were mostly round, oval, nodular or lobular, with wide base, smooth or uneven surface, medium hardness, complete envelope, clear boundary, poor mobility, and easy palpation bleeding.

The radiographic findings of pleomorphic adenomas in the nasal cavity are nonspecific, much like the clinical presentation. CT usually shows a clear soft tissue mass; Calcification.

occurs only in rare cases.The clinician can evaluate bone involvement or destruction based on CT, which may occur when a neoplasm has been untreated for a long time [10].

Magnetic Resonance Imaging (MRI) is primarily used to evaluate the epithelial and stromal components of tumors, as well as the surrounding soft tissues. MRI of interstitial components showed T1-weighted low signal intensity and T2-weighted medium-high signal intensity. Epithelial components have low signal intensity on T2-weighted imaging [6]. Kajiyama et al. [11] found that MRI of nasal polymorform adenoma mainly showed submucosa lesion, and suggesting that preoperative MRI examination should be performed to ensure the complete resection of the tumor.

Diagnosis of Pleomorphic Adenoma mainly relies on pathologic examination of the mass [2–4, 6].

At low magnification, the outer cells of the lumen were arranged in palisades, nests, sheets or strips, with obvious proliferation and abundant lumen structure. At high magnification, tumor myoepithelial cells were mixed with epithelial cells, or are arranged like spindle, plasma cells, clear cells, and epithelial cells. Unlike the pleomorphic adenoma of the large salivary gland, the PANS contain more cellularity; predominant epithelial components; Low stromal component and absent capsule.

Immunohistochemistry can helpful in the diagnosis of pleomorphic adenoma: [2, 4] Ki-67 is used to judge the proliferation activity of cells, and previous studies have shown that the positive expression rate of Ki-67 in pleomorphic adenoma of the nose is 1% ~ 5%. S-100, P63, CK (AE1/AE3) and SMA are all sensitive markers of myoepithelial cells, and studies have shown that they are all positive in pleomorphic adenoma of nose.

Differential diagnosis of intranasal pleomorphic adenoma includes both benign and malignant tumours such as polyps, capillary tumor, papillomas, angiofibromas, myoepithelioma, retention cyst, osteomas, squamous cell carcinoma, mucoepidermoid carcinoma, adenocarcinoma, adenoid cystic carcinoma, melanoma and olfactory esthesioneuroblastoma [12].

Surgical treatment is the preferred treatment for nasal pleomorphic adenomas [6, 8]. The goal is to preserve a safe margin, remove the tumor completely, and prevent metastasis of tumor cells through blood or lymph node implantation [13]. Different surgical methods can be used according to tumor growth site and size. It include endonasal endoscopic resection, midfacial degloving approach, lateral rhinotomy, and partial maxillectomy. [2, 14] Endoscopic resection of this type of tumor has become the preferred surgical method for this tumor due to its advantages of less damage, less damage to the tumor envelope, better protection of the normal structure of the nasal cavity, and better recovery of the physiological function of the nasal cavity after surgery [15].

However, surgical indications should be considered in endoscopic resection of septum pleomorphic adenoma.

If the nasal tumor is large, poorly defined, or suspected of malignant metastasis, rhinotomy is recommended to avoid recurrence or tumor tumor rupture due to incomplete or insufficient resection [8, 16].

There are reports that malignant transformation of PNAS is still very rare, with fewer than 10 cases [17]. The incidence has been reported in 2.4 to 10% of cases [14]. The reasons for recurrence are may be as follows:

(1) Partial tumor tissue capsule adheres to normal nasal mucosa, which is not completely removed during the operation, resulting in residual tumor tissue.

(2) Tumor rupture, tumor tissue overflow and lead to implant recurrence.

Therefore, Freeman et al. [18] suggested extending local resection to ensure the integrity of tumor capsule so as to prevent local and distant implantation of tumor cells. At the same time, if the tumor is suspected to be preoperatively, routine biopsy should not be performed to prevent capsule rupture, and rapid frozen sections can be performed intraoperatively. In order to completely remove the tumor, an incision should be made in the normal tissue outside the tumor boundary. If the tumor is found to be broken intraoperatively, the tumor tissue should be carefully removed, and the normal saline should be repeatedly rinsed and the surgical instruments replaced [16].

Pleomorphic adenomas do not respond well to chemotherapy and radiotherapy. In addition, radiation may induce malignant transformation of tumors [19]. Therefore, it is generally not recommended. However, it has also been reported that postoperative adjuvant radiotherapy can reduce the recurrence rate of positive surgical margin and polynodular pleomorphic adenoma [16, 20].

## Conclusion

pleomorphic adenoma of nasal septum is a rare benign tumor with no specificity in imaging diagnosis. Patients are usually diagnosed when they come to the hospital with unilateral nasal congestion or progressive nasal bleeding. and pathological diagnosis is the gold standard for this disease.

The possibility of a pleomorphic adenoma of nasal cavity should be considered when a single round smooth tumor is clinically seen, especially originated from nasal septum. A complete resection of the tumor along the tumor base boundary beyond 5 mm is preferred with low temperature plasma knif under the nasal endoscopy. Postoperative pathological biopsy and immunohistochemical detection are performed to confirm the diagnosis. Long-term follow-up after surgery is recommended because of the risk of recurrence of the tumor.

## Abbreviations

PANS: Pleomorphic adenoma of nasal septum
CT: Computed tomographic scans
MRI: Magnetic Resonance Imaging

## References

[1] Tahlan A, Nanda A, Nagarkar N, Bansal S (2004). Pleomorphic adenoma of the nasal septum: a case report. Am J Otolaryngol.

[2] Bose S, Agarwal M, Nawale K (2020). Pleomorphic adenoma of the nasal septum - A rare entity. Natl J Maxillofac Surg.

[3] Baglam T, Durucu C, Cevik C, Bakir K, Oz A, Kanlikama M (2011). Giant pleomorphic adenoma of the nasal septum. Indian J Otolaryngol Head Neck Surg.

[4] Hirai S, Matsumoto T, Suda K (2002). Pleomorphic adenoma in nasal cavity: immunohistochemical study of three cases. Auris Nasus Larynx.

[5] Sadeghi EM, Darling JE (1994). Cystic pleomorphic adenoma of the mandible. Int J Oral Maxillofac Surg.

[6] Acevedo JL, Nolan J, Markwell JK, Thompson D (2010). Pleomorphic adenoma of the nasal cavity: a case report. Ear Nose Throat J.

[7] Compagno J, Wong RT (1977). Intranasal mixed tumors (pleomorphic adenomas): a clinicopathologic study of 40 cases. Am J Clin Pathol.

[8] Rha M, Jeong S, Cho H, Yoon J, Kim C (2018) Sinonasal pleomorphic adenoma: a single institution case series combined with a comprehensive review of literatures.AURIS NASUS LARYNX. 46(2) 10.1016/j.anl.2018.08.003 30122650

[9] Gana P, Masterson L (2008). Pleomorphic adenoma of the nasal septum: a case report. J Med Case Rep.

[10] Clark M, Fatterpekar GM, Mukherji SK, Buenting J (1999). CT of intranasal pleomorphic adenoma. Neuroradiology.

[11] Akiko K, Hiromi E, Natsuki I, Yuki Y, Tatsuya G (2019) Magnetic resonance imaging and histopathology in a case of Pleomorphic Adenoma of a minor salivary gland in the nasal cavity.American Journal of Case Reports.20 10.12659/AJCR.915491 PMC6530521 31079138

[12] Shetty S, Nayak DR, Jaiprakash P (2018) Pleomorphic adenoma of nasal septum: a rare case.BMJ Case Reports. 2018(mar28 1). 10.1136/bcr-2017-223148 PMC5878320 29592986

[13] Ceylan A, Celenk F, Poyraz A, Uslu S (2008). Pleomorphic adenoma of the nasal columella. Pathol Res Pract.

[14] Fushiki H, Morijiri M, Maruyama M, Motoshima H, Watanabe Y (2006). MRI of intranasal pleomorphic adenoma. Acta Otolaryngol.

[15] Shetty S, Nayak DR, Jaiprakash P (2018) Pleomorphic adenoma of nasal septum: a rare case. BMJ Case Rep. 2018 10.1136/bcr-2017-223148 PMC5878320 29592986

[16] Bradley PJ (2018). The recurrent pleomorphic adenoma conundrum. Curr Opin Otolaryngol Head Neck Surg.

[17] Li W, Lu H, Zhang H et al (2019) Sinonasal/nasopharyngeal pleomorphic adenoma and carcinoma ex pleomorphic adenoma: a report of 17 surgical cases combined with a literature review.Cancer Management and Research. Volume 11 10.2147/CMAR.S198942 PMC6588090 31354359

[18] Freeman SB, Kennedy KS, Parker GS, Tatum SA (1990). Metastasizing pleomorphic adenoma of the nasal septum. Arch Otolaryngol Head Neck Surg.

[19] Saksela E, Tarkkanen J, Kohonen A (1970). The malignancy of mixed tumors of the parotid gland. A clinicopathological analysis of 70 cases. Acta Otolaryngol.

[20] Renehan A, Gleave EN, McGurk M (1996) An analysis of the treatment of 114 patients with recurrent pleomorphic adenomas of the parotid gland.The American Journal of Surgery. 172(6) 10.1016/s0002-9610(96)00293-0 8988685

